# Metabolic functions of macropinocytosis

**DOI:** 10.1098/rstb.2018.0285

**Published:** 2018-12-17

**Authors:** Wilhelm Palm

**Affiliations:** German Cancer Research Center (DKFZ), Im Neuenheimer Feld 280, 69120 Heidelberg, Germany

**Keywords:** macropinocytosis, lysosome, nutrients, cancer metabolism, growth factors, mTORC1

## Abstract

Macropinocytosis is an evolutionarily conserved form of endocytosis that mediates non-selective uptake of extracellular fluid and the solutes contained therein. In mammalian cells, macropinocytosis is initiated by growth factor-mediated activation of the Ras and PI3-kinase signalling pathways. In malignant cells, oncogenic activation of growth factor signalling sustains macropinocytosis cell autonomously. Recent studies of cancer metabolism, discussed here, have begun to define a role for macropinocytosis as a nutrient uptake route. Macropinocytic cancer cells ingest macromolecules in bulk and break them down in the lysosome to support metabolism and macromolecular synthesis. Thereby, macropinocytosis allows cells to tap into the copious nutrient stores of extracellular macromolecules when canonical nutrients are scarce. These findings demonstrate that macropinocytosis promotes metabolic flexibility and resilience, which enables cancer cells to survive and grow in nutrient-poor environments. Implications for physiological roles of growth factor-stimulated macropinocytosis in cell metabolism and its relationship with other nutrient uptake pathways are considered.

This article is part of the Theo Murphy meeting issue ‘Macropinocytosis’.

## 1. Introduction

Macropinocytosis was first described in the early twentieth century, when microscopy studies revealed that some mammalian cells constantly internalize surrounding fluid into large vesicles [1]. In accordance with the previously discovered phagocytosis or cell eating, which refers to the engulfment of bacteria and solid particles, the process of swallowing extracellular fluid was named pinocytosis or cell drinking. Later on, the term macropinocytosis was coined to distinguish fluid-phase uptake into large vesicles from various small-scale pinocytic pathways. Macropinocytosis is now recognized as an evolutionarily conserved form of endocytosis that occurs in diverse cell types, for example in amoebae, *Drosophila* haemocytes and diverse mammalian cells including monocytes, fibroblasts, epithelial cells and neurons [2–4]. Moreover, macropinocytosis since its discovery has been recognized as a feature commonly associated with malignant cells [5].

In mammals, functions of macropinocytosis are best understood in immunity and infection [2–4]. Owing to its non-selective nature, macropinocytosis allows macrophages and immature dendritic cells to sample their environment for soluble antigens that are presented to T cells. The large size of macropinosomes is exploited by some viruses and bacteria as an entry route to invade host cells. Another proposed function is rapid remodelling of the plasma membrane composition by internalization of large membrane patches into macropinosomes. This mechanism facilitates the redistribution of integrins during cell migration and could allow adjustment of the plasma membrane components of signal transduction pathways [6,7]. In most cell types, however, physiological roles of macropinocytosis have been unclear.

**Figure 1.**
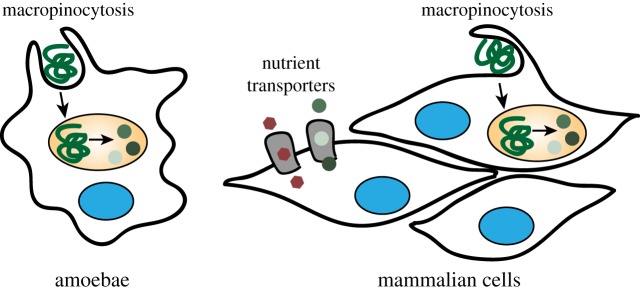
Nutrient uptake strategies of eukaryotic cells. Unicellular eukaryotes such as the amoeba *Dictyostelium discoideum* feed by ingesting macromolecular nutrients and bacteria through macropinocytosis or the related phagocytosis. Subsequently, the macromolecular foodstuff is broken down in the lysosome to generate an intracellular nutrient source. Mammalian cells usually reside in an environment that provides ample amino acids and saccharides, which cells import through plasma membrane nutrient transporters. However, macropinocytosis and the endolysosomal system are conserved in mammalian cells and have emerged as a pathway to access the nutritional content of extracellular macromolecules.

**Figure 2.**
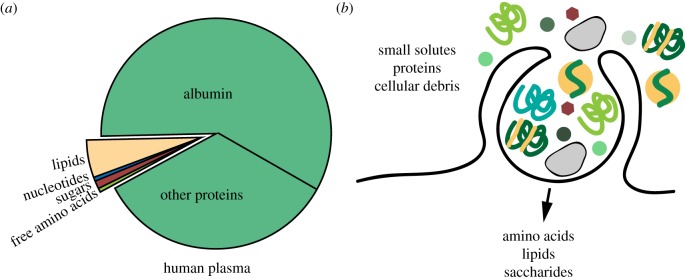
Metabolic benefits of macropinocytosis. (*a*) Biomass composition of human plasma. Of note, most biomass in circulation is contained within proteins. (*b*) Macropinocytosis internalizes nutrients according to their extracellular concentration. Proteins are a major cargo of macropinosomes and supply amino acids, and possibly bound lipids, sugar residues and micronutrients. Cellular debris, which is abundant in some pathological contexts, can also be ingested through macropinocytosis.

The study of unicellular eukaryotes such as the social amoeba *Dictyostelium discoideum* has identified a metabolic role for macropinocytosis—the uptake of macromolecular nutrients [2]. Upon trafficking of macropinosomes to the lysosome, organelle-resident hydrolases break down the macromolecular cargo into their monomeric constituents, thereby creating an intracellular source of diverse nutrients (figure 1). In animals, the intestine digests dietary macromolecules and releases their monomeric building blocks, sugars, amino acids and lipids, into circulation as nutrients for other cells in the body. This led to the notion that mammalian cells use transmembrane transporters rather than endocytosis to acquire bulk nutrients. However, most biomass in the extracellular space is contained within macromolecules, which have the potential to function as important nutrients, if accessible to cells (figure 2*a*). Recently, the study of cancer metabolism has demonstrated that some transformed cells exploit macropinocytosis to non-selectively ingest and digest macromolecules, and thereby survive and grow in nutrient-poor tumour environments [8–10]. The analogous roles of macropinocytosis in unicellular eukaryotes and cancer cells suggest uptake of macromolecular nutrients as its ancestral and evolutionarily conserved function. Almost a century after cell drinking was described in mammalian cells, it is now aptly recognized as a nutrient uptake pathway.

## 2. The process of macropinocytosis

Macropinocytosis is a non-selective endocytic pathway that internalizes extracellular fluid and therein contained solutes into large vesicles called macropinosomes [2–4]. Macropinocytosis is initiated by actin-driven protrusions of the plasma membrane that form cup-shaped ruffles. When such membrane ruffles fold back, they enclose portions of extracellular fluid and through closure and pinching off from the plasma membrane give rise to irregularly shaped vesicles of varying size. Owing to its non-selective nature, macropinocytosis in principle allows cells to ingest any soluble macromolecule from their environment. Owing to their large size, macropinosomes can accommodate cargo that is excluded from other endocytic vesicles, including diverse macromolecules and even cell-derived particles such as exosomes, apoptotic bodies and cellular debris [10] (figure 2*b*). Once formed, macropinosomes can have two intracellular fates: recycling to the cell surface for cargo release back into the extracellular space, or trafficking to the lysosome, which contains hydrolytic enzymes—proteases, lipases, glycosidases and nucleases—that break down the macromolecular cargo [11,12].

Mechanistically, macropinocytosis most closely resembles phagocytosis: both endocytic pathways are initiated by actin-driven protrusions of the plasma membrane [3]. However, a distinguishing mark of macropinocytosis is its induction in the absence of cargo. By contrast, phagocytosis is initiated by solid particles or bacteria, which are enclosed by plasma membrane extensions, and receptor-mediated endocytosis is triggered by binding of macromolecules to cognate cell surface receptors. The regulators of subcellular macropinosome trafficking are shared with other endocytic pathways [4,13]. Macropinosomes become rapidly decorated with Rab5, which is substituted with Rab7 during their maturation and lysosomal trafficking. During this process, maturing macropinosomes acquire content and components of late endosomes and lysosomes [11,14]. As a consequence, macropinosomes are readily recognized by virtue of their large size, but their identification on the basis of molecular markers is less clear.

## 3. Regulation of macropinocytosis by growth factors and nutrient sensors

**Figure 3.**
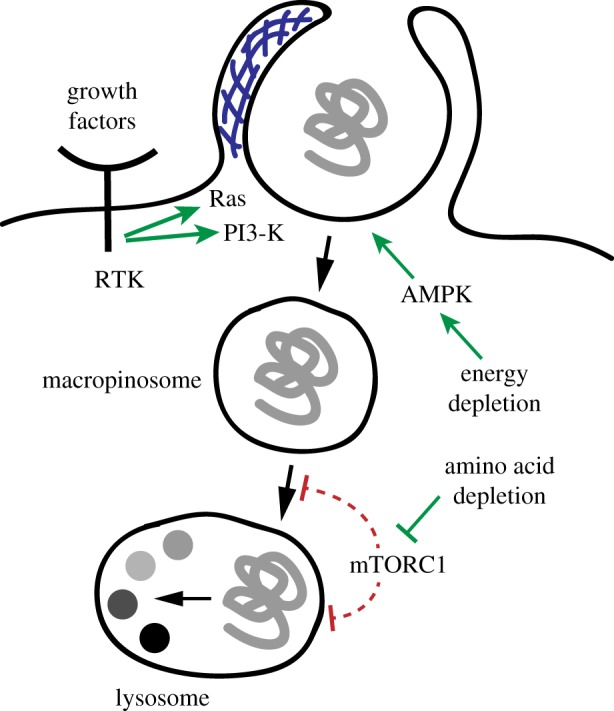
Regulation of macropinocytosis. Macropinocytosis is controlled by growth factor signalling. Growth factors activate their cognate receptor tyrosine kinases (RTKs), which through their effectors Ras and PI3-kinase orchestrate actin-driven membrane ruffling and macropinosome formation. Oncogenic mutations that constitutively activate the Ras and PI3-kinase signalling pathways trigger macropinocytosis cell autonomously. Macropinocytosis and lysosomal catabolism of extracellular proteins is also regulated by the cellular metabolic state. Energy depletion leads to activation of AMPK, which in some contexts promotes macropinocytosis induction. Amino acid depletion leads to the inactivation of mTORC1, which enhances lysosomal catabolism of macropinocytosed proteins.

Unlike other endocytic pathways, macropinocytosis is acutely induced by growth factors (figure 3) [3,4]. In fact, membrane ruffling and macropinosome formation are among the earliest cellular responses to growth factor stimuli [15,16]. The signal transduction cascade that initiates macropinosome formation is centred around the small GTPase Ras and Class I phosphatidylinositol 3-kinase (PI3-kinase). Genetic experiments in *Dictyostelium* suggest that Ras acts as the proximal signal, which induces plasma membrane recruitment and activation of PI3-kinase [2]. Once active, Ras and PI3-kinase signal through several effectors including the small GTPases Rac1 and Rab5 and the kinase PAK1 to orchestrate actin polymerization and membrane ruffling as well as the other molecular events that contribute to macropinosome formation and closure [3,17–19]. In mammalian cells, Ras and PI3-kinase are activated by receptor tyrosine kinases such as epidermal growth factor receptor and platelet-derived growth factor receptor. Inhibiting receptor tyrosine kinases or PI3-kinase blocks macropinocytosis [20–22]. By contrast, deletion of all major Ras isoforms in fibroblasts does not abrogate growth factor-stimulated macropinocytosis [23]. Like its *Dictyostelium* homologue, mammalian PI3-kinase has a Ras-binding domain, which contributes to its recruitment to the plasma membrane. However, plasma membrane recruitment and activation of mammalian PI3-kinase is orchestrated by receptor tyrosine kinases and can occur independently of Ras. This may explain why Ras triggers macropinocytosis but is not strictly required for its induction by external growth factor stimuli.

Trafficking of macropinosomes to the lysosome is a committed step at which cells decide whether ingested macromolecules are broken down into their building blocks (figure 3). The nutrient-sensing kinase mechanistic target of rapamycin complex 1 (mTORC1) is a central regulator of this process. mTORC1 is activated by concerted inputs from intracellular amino acids and growth factor signalling and in turn promotes biosynthetic pathways while blocking macromolecular catabolism [24,25]. Inhibition of mTORC1 causes a rapid and substantial increase in lysosomal catabolism of proteins ingested from the environment [26]. However, mTORC1 does not act at the step of macropinosome formation. Rather, mTORC1 appears to suppress trafficking of macropinosomes to the lysosome or hydrolytic activity of the lysosome through yet to be identified mechanisms. In addition, amino acid deficiencies upregulate lysosomal catabolism of extracellular proteins in an mTORC1-independent process [27]. As a consequence, macropinocytic cells catabolize proteins only inefficiently when amino acids are abundant and mTORC1 is active. These mechanisms presumably operate in nutrient-rich environments to prevent cells from engaging in the wasteful catabolism of extracellular proteins. While mTORC1 signals under nutrient abundance, AMP-activated protein kinase (AMPK) orchestrates cellular responses to energy shortage [28]. In cells depleted of PTEN, a negative regulator of PI3-kinase signalling, AMPK activates Rac1, thereby promoting macropinosome formation [29]. AMPK also antagonizes the mTORC1 pathway and therefore conceivably enhances the efficiency at which macropinocytosed proteins are catabolized in the lysosome. The responsiveness of macropinocytosis and lysosomal catabolism of extracellular proteins to metabolic cues suggests that this endocytic pathway is part of the cellular adaptation to starvation.

While macropinocytosis is regulated by signal transduction, it reciprocally influences cell signalling. Amino acids either imported through plasma membrane transporters or ingested with extracellular fluid through macropinocytosis can activate mTORC1 [30]. While nutrient transporters flux amino acids into the cytosol at high rates, macropinocytic uptake of extracellular fluid conceivably contributes to intracellular amino acid pools in contexts where transporter capacity is limiting. By supplying extracellular proteins to the lysosome and generating an intracellular amino acid source, macropinocytosis also sustains mTORC1 activity in cells that reside in protein-rich but amino acid-poor environments [23]. Of note, mTORC1 is regulated by cytosolic as well as lysosomal amino acid sensors [31], which could differ in their sensitivities to amino acids taken up through plasma membrane transporters or macropinocytosis.

## 4. Macropinocytosis induction by oncogenes

Macropinocytosis is a common feature of many cancer cells [5]. The molecular basis for the close association between cellular transformation and macropinocytosis lies in the prevalence of oncogenic mutations in several of the core signalling components that regulate macropinosome formation. In particular, activating mutations in Ras initiate macropinocytosis independently of growth factor stimulation [20,32]. Oncogenic mutations in receptor tyrosine kinases or PI3-kinase as well as autocrine signalling loops by cancer cells that secrete growth factors also have the potential to promote macropinocytosis. The fundamental trait of transformed cells to cell autonomously sustain proliferative signalling is thus closely associated with high macropinocytic activity.

Besides receptor tyrosine kinases, Ras and PI3-kinase, other components of growth factor signalling pathways that regulate macropinocytosis are mutated in cancer. PTEN, the phosphatase antagonizing PI3-kinase signalling, is a major tumour suppressor and its deletion promotes macropinocytosis [23]. Ras GTPase activating proteins function as a tumour suppressor through negative regulation of Ras; epigenetic silencing of RASAL3 increases Ras signalling and macropinocytosis, and deletion of NF1, which is common in cancer, conceivably also enhances macropinocytosis [33,34]. Interestingly, *Dictyostelium* strains harbouring deletions in NF1 display increased macropinocytosis, which allows them to grow by feeding on extracellular proteins [35]. Moreover, cancer-associated mutations or amplifications occur in Rac1 and PAK1, both regulators of actin polymerization and membrane ruffling downstream of Ras and PI3-kinase [36–38]. Macropinocytosis has also been identified as a novel function of several other signalling pathways that contribute to carcinogenesis: the tumour suppressor NF2 negatively regulates growth factor-induced macropinocytosis [39]. Canonical Wnt signalling was identified in a genome-wide screen for macropinocytosis activators in colon cancer [40]. It would be unsurprising if ongoing efforts to dissect the dysregulation of macropinocytosis in cancer cells led to the identification of further oncogenes or tumour suppressors involved in this process.

## 5. Macropinocytosis in cancer metabolism

Despite the correlation of macropinocytosis with oncogenic signal transduction, the functional relevance of this phenomenon has only recently begun to emerge from the study of cancer metabolism. Cancer is essentially a disease of dysregulated cell proliferation. However, solid tumour growth disrupts the orderly organization of healthy tissue and creates extensive regions with poor or abnormal vasculature, which are nutrient-deprived. Therefore, cancer cells face two metabolic challenges—the fast growth rates of transformed cells create high nutritional demands, while the fluctuating nutrient supply in tumours creates metabolic stresses [41,42]. To support anabolic metabolism and biomass formation, cancer cells select for mutations that promote nutrient uptake. To survive and proliferate in harsh tumour microenvironments, cancer cells select for mutations that enhance metabolic flexibility and resilience. For instance, many cancer cells take up large quantities of glucose and glutamine but can oftentimes compensate for shortages of either nutrient by increased usage of the other [41,42]. To this end, carbons derived from either glucose or glutamine can be used for tricarboxylic acid cycle anaplerosis or substituted by increased catabolism of other amino acids.

Besides flexibility in glucose and amino acid metabolism, some cancer cells have an altogether different solution for coping with nutrient deficiencies—macropinocytosis of extracellular macromolecules as a non-canonical nutrient source. The first evidence for macropinocytosis as a nutrient uptake pathway came from the study of pancreatic cancer cells, which harbour oncogenic K-Ras alleles and are macropinocytic, and fibroblasts genetically engineered to constitutively activate macropinocytosis [26,43,44]. These experiments demonstrated that macropinocytosis and lysosomal catabolism of extracellular proteins creates an intracellular nutrient source. Amino acids derived from macropinocytosed proteins supply carbons to central metabolism and are used for protein synthesis, which supports cell proliferation under conditions where free amino acids are depleted extracellularly. Physiological levels of albumin, the major protein in circulation, enable pancreatic cancer cells to proliferate under limiting glutamine concentrations [44]. Albumin catabolism even supports the proliferation of various cancer cells as well as fibroblasts expressing oncogenic Ras or PI3-kinase mutants in the complete absence of essential amino acids such as leucine [23,26,27,43]. Upon adaptation to albumin as an obligatory nutrient, some cancer cells can grow with one population doubling per day—a proliferation rate not uncommon for cells that grow in nutrient-rich media—demonstrating the efficiency at which the endolysosomal system can recover amino acids from extracellular proteins [27].

Albumin as the major protein in circulation has been the focus of studies investigating how cancer cells co-opt macropinocytosis to acquire amino acids. However, the extracellular matrix represents a major fraction of extracellular biomass in tissues. Tumours commonly display elevated levels of extracellular proteolysis [45] and macropinocytosis could conceivably allow cancer cells to ingest fragments of extracellular matrix proteins generated during this process. Necrotic regions of a solid tumour contain cell debris, which can be taken up through macropinocytosis and sustain cancer cell survival during nutrient deprivation [29]. Besides amino acids, the diverse macromolecules and macromolecular particles present in tumour microenvironments could also supply other nutrients such as lipids and sugars.

The discovery of macropinocytosis as a nutrient acquisition pathway in cancer cells establishes a novel function for the oncogenes and tumour suppressors that comprise growth factor signalling pathways. By allowing access to the nutritional contents of extracellular macromolecules, oncogenic activation of macropinocytosis grants cancer cells metabolic flexibility and resilience to endure under conditions that do not support survival and growth of non-transformed cells [23,26,29,43,44]. Oncogenic mutations in the Ras and PI3-kinase signalling pathways not only increase glucose uptake, which contributes to the Warburg effect, but also trigger macropinocytosis, which allows utilization of macromolecular nutrients [10,46]. Thus, the oncogenic potential of Ras and PI3-kinase signalling may lie, in part, in their ability to induce constitutive macropinocytosis. By contrast, mTORC1 suppresses the use of proteins as nutrients and therefore limits a cell's metabolic flexibility. Inhibition of mTORC1 enhances lysosomal catabolism of macropinocytosed proteins, which substantially increases the proliferation of cancer cells that rely on extracellular proteins as an amino acid source [26]. Consistently, activating mTOR mutations occur in cancer at comparably low frequencies, and loss of the negative mTORC1 regulators TSC1/2 are mainly found in tumours that arise in well-perfused organs such as brain, heart and kidney [47,48].

The first *in vivo* clues for the importance of extracellular proteins as nutrients for cancer cells came from the observation that rat carcinoma accumulates high levels of radioactively labelled albumin [49]. While the cellular pathway of albumin uptake was not identified, this study indicated that albumin was taken up by tumour tissue and catabolized in the lysosome. More recently, uptake of high-molecular-weight dextran by cancer cells was detected *in vivo* in pancreatic tumours from mice and human patients, providing direct evidence for macropinocytic activity in cancer cells [43,44]. Moreover, a sophisticated approach using tissue microimplants that release fluorescent macromolecules allowed the monitoring of macropinocytosis as well as albumin catabolism in murine pancreatic tumours [50]. Together, these experiments demonstrated elevated macropinocytosis and albumin catabolism in tumours as compared to adjacent non-cancerous tissue, confirming earlier observations in cultured cancer cell lines. Plasma protein catabolism by tumours is also consistent with metabolite levels in tumour samples from human patients, which display an enrichment of essential amino acids but depletion of non-essential amino acids [43]. Such imbalances could arise when tumour cells ingest and catabolize extracellular proteins and preferentially metabolize their non-essential amino acid content.

## 6. Does macropinocytosis play a physiological role in nutrient uptake?

Cancer cells commonly exploit physiological processes, for instance, by constitutive activation or hyper-activation of regulatory circuits. This raises the possibility that macropinocytosis and lysosomal catabolism of extracellular macromolecules plays a physiological role in cellular nutrient uptake that is co-opted by cancer. While metabolic functions of macropinocytosis in non-pathological contexts largely remain to be investigated, two considerations suggest their importance: most extracellular biomass is contained within macromolecules, and their cellular uptake through macropinocytosis is initiated by growth factors.

The proteins and proteoglycans present in the circulation and the extracellular matrix exert diverse functions, such as mechanical stability, metabolite transport and signalling. However, their common feature may be less appreciated—macromolecules contain most extracellular biomass and thus constitute vast, non-dedicated nutrient stores. As a fluid-phase endocytic pathway, macropinocytosis allows cells to access the nutritional content of diverse extracellular macromolecules, which could supply a range of nutrients, including amino acids, lipids, sugars and nucleotides. Albumin, for instance, is the major protein in circulation, functions in oncotic pressure regulation and fatty acid transport, but also contains amino acids exceeding free amino acid concentrations in plasma by several hundred fold [51]. Macropinocytosis and catabolism of but a small fraction of albumin would therefore generate amino acids in substantial quantities. When free amino acids are scarce and mTORC1 activity declines, macropinocytosis of albumin and other extracellular proteins could thus sustain cellular homeostasis. This process is exploited by nutrient-deprived cancer cells but could also pertain to other contexts where nutrient deficiencies arise, for instance, in tissues where vascular supply is compromised [8–10].

Growth factors regulate nutrient uptake in mammalian cells—they promote expression and surface presentation of various glucose and amino acid transporters and upregulate endocytic receptors for circulating nutrient carriers such as low-density lipoproteins and transferrin [10,46]. The cell-extrinsic control of nutrient uptake ensures that nutrient supply matches the metabolic demands of growth and prevents excessive consumption of metabolic resources. Macropinocytosis induction is an immediate cellular response to growth factor stimulation and is orchestrated by the same pathways that regulate other nutrient uptake routes—the Ras and PI3-kinase signalling pathways. Thus, macropinocytosis could be part of the metabolic programme by which growth factors support biomass formation and growth.

## 7. Relationship to other nutrient uptake pathways

**Figure 4.**
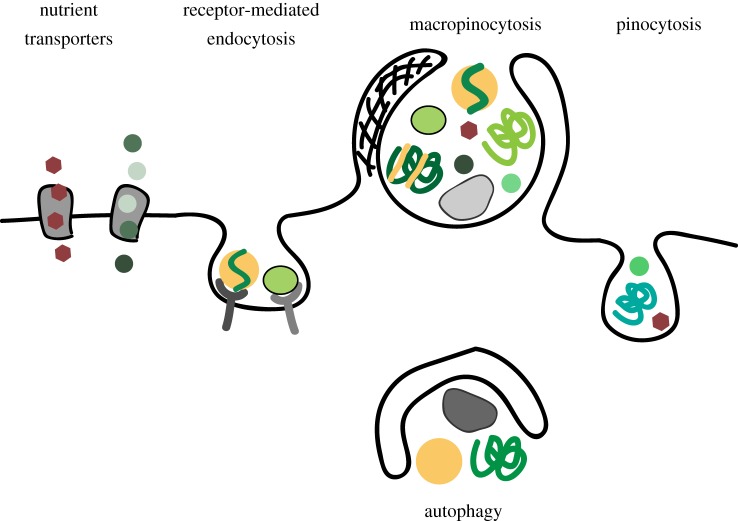
Macropinocytosis and other nutrient uptake pathways. Nutrient transporters and endocytic receptors mediate selective uptake of dedicated nutrients such as glucose, amino acids and lipoproteins. Macropinocytosis allows cells to access non-dedicated nutrient stores of extracellular macromolecules, with a possible contribution from constitutive pinocytosis. Autophagy has an analogous role in mediating breakdown of intracellular macromolecules as non-dedicated nutrients.

As discussed above, nutrient uptake through macropinocytosis is exploited by cancer cells and conceivably contributes to the metabolism of normal cells. What then is its functional relationship with other nutrient acquisition pathways? Nutrient transporters flux glucose and amino acids across the plasma membrane, and endocytic receptors mediate selective uptake of carrier proteins that transport water-insoluble nutrients such as lipids and iron (figure 4). By contrast, macropinocytosis as a fluid-phase endocytic pathway internalizes solutes according to their extracellular concentration. Therefore, proteins as the major fraction of circulating biomass presumably constitute the major macropinosome cargo *in vivo*. Concomitantly, cells macropinocytose those molecules associated with proteins such as albumin-bound fatty acids [52]. Thereby, macropinocytosis conceivably supplies lipids, vitamins and metal ions, some of which may not be accessible by other means. However, due to its non-selective nature, macropinocytosis is likely not particularly efficient in concentrating nutrients that are of low abundance or required in large quantities. Mammalian cells also engage in constitutive pinocytosis, which constantly internalizes extracellular fluid into small endocytic vesicles and is active in quiescent cells [13]. Whether pinocytosis exerts metabolic functions or is exploited by cancer cells for nutrient uptake is largely unknown, but this pathway could supply extracellular proteins in quiescent cells, which do not macropinocytose.

Several aspects of macropinocytosis bear functional resemblance to macroautophagy, hereafter referred to as autophagy, a vesicular pathway that mediates breakdown of intracellular macromolecules [53,54] (figure 4). During autophagy, cytosolic constituents and organelles are engulfed by double-membrane vesicles called autophagosomes, which are subsequently degraded in lysosomes. Similar to macropinocytosis, autophagy can non-selectively deliver diverse macromolecules as well as macromolecular assemblies and organelles to the lysosome. Macropinocytosis and autophagy allow cells to tap into the copious nutritional stores of extra- and intracellular macromolecules, respectively. Cancer cells exploit both pathways to supply macromolecules to lysosomal catabolism, thereby sustaining survival and growth in harsh tumour microenvironments where nutrient levels fluctuate [26,43,44,55,56]. Autophagy also plays a central role in cellular adaptation to starvation in healthy tissue; the contribution of macropinocytosis to metabolic homeostasis during starvation in physiological contexts remains to be defined.

Like nutrient recovery through macropinocytosis, autophagy is regulated by nutrient-sensing signalling pathways: autophagosome formation is blocked by mTORC1 and initiated by AMPK [28]. Thus, under nutrient abundance, non-selective catabolism of macromolecules from both internal and external sources is suppressed. When nutrient levels decline, cells upregulate lysosomal catabolism of macromolecules derived from extracellular and intracellular sources. Aspects of macropinocytosis and autophagy are also coordinated by other processes, for instance, microRNA-mediated co-regulation [57]. However, macropinocytosis differs from autophagy in its regulation by growth factors. Consistently, growth factor-induced macropinocytosis supplies exogenous nutrients, which can support continuous cell growth under amino acid starvation. By contrast, autophagy is activated by growth factor withdrawal and can support cell survival only during limited periods of starvation, but eventually results in cellular atrophy [58].

Besides its metabolic function during starvation, autophagy contributes to cellular quality control by degrading dispensable or damaged macromolecules and organelles [54]. In an interesting analogy, when macropinocytosis was discovered, one of its proposed functions was to keep tissue juices in proper condition by removing extracellular waste [5]. Removal of damaged cells and cellular debris is now known to be mainly performed by professional phagocytes. However, macropinocytosis can mediate engulfment of apoptotic and necrotic bodies by various cell types [21,29,59]. Conceivably, this could contribute to quality control in the extracellular space when immune cells get overwhelmed.

## 8. Future directions

Macropinocytosis has emerged as a feeding strategy through which cancer cells access the nutrient stores of extracellular macromolecules to support survival and growth in nutrient-deprived tumour microenvironments. Ras and PI3-kinase are major oncogenes with well-established roles in the regulation of macropinosome formation. An increasing number of additional cancer-associated mutations are implicated in the regulation of macropinocytosis and lysosomal catabolism. Moving forward, it will be important to clarify to what extent macropinocytosis induction contributes to the cancer-promoting action of these mutations. In addition, it remains challenging to determine in specific tumour contexts the nutrients supplied by macropinocytosis, as well as relative contributions of macropinocytosis and other nutrient acquisition pathways to cancer metabolism.

Understanding oncogenic roles of macropinocytosis further provides a conceptual framework in which to interrogate its physiological functions in cell metabolism. Constitutive macropinocytosis allows macrophages and dendritic cells to take up antigens but could also supply nutrients to support immune cell function, for instance, in tumour microenvironments. Macropinocytosis could also allow other cells to use the nutritional content of extracellular macromolecules to buffer fluctuations in the supply of amino acids and other nutrients. In this context, the relative contribution of macropinocytosis and autophagy remains to be addressed. This is particularly important because cellular starvation responses are routinely investigated under culture conditions where extracellular macromolecules are depleted, which may underestimate the metabolic role of macropinocytosis. Lastly, it remains puzzling why growth factors stimulate macropinocytosis under nutrient-rich conditions, where catabolism of extracellular macromolecules is energetically wasteful and hence suppressed. Perhaps growth factor-initiated macropinocytosis ensures uptake of the diverse micronutrients required for biomass formation, for which more selective uptake mechanisms might not operate.

## Data accessibility

This article has no additional data.
